# Novel Pannexin-1-Coupled Signaling Cascade Involved in the Control of Endothelial Cell Function and NO-Dependent Relaxation

**DOI:** 10.1155/2021/2678134

**Published:** 2021-02-20

**Authors:** Mauricio A. Lillo, Pablo S. Gaete, Mariela Puebla, Pía C. Burboa, Inés Poblete, Xavier F. Figueroa

**Affiliations:** ^1^Departamento de Fisiología, Facultad de Ciencias Biológicas, Pontificia Universidad Católica de Chile, Santiago 8330025, Chile; ^2^Centro de Fisiología Celular e Integrativa, Facultad de Medicina-Clínica Alemana, Universidad del Desarrollo, Santiago, Chile

## Abstract

Deletion of pannexin-1 (Panx-1) leads not only to a reduction in endothelium-derived hyperpolarization but also to an increase in NO-mediated vasodilation. Therefore, we evaluated the participation of Panx-1-formed channels in the control of membrane potential and [Ca^2+^]_i_ of endothelial cells. Changes in NO-mediated vasodilation, membrane potential, superoxide anion (O_2_^·–^) formation, and endothelial cell [Ca^2+^]_i_ were analyzed in rat isolated mesenteric arterial beds and primary cultures of mesenteric endothelial cells. Inhibition of Panx-1 channels with probenecid (1 mM) or the Panx-1 blocking peptide ^10^Panx (60 *μ*M) evoked an increase in the ACh (100 nM)-induced vasodilation of KCl-contracted mesenteries and in the phosphorylation level of endothelial NO synthase (eNOS) at serine 1177 (P-eNOS^S1177^) and Akt at serine 473 (P-Akt^S473^). In addition, probenecid or ^10^Panx application activated a rapid, tetrodotoxin (TTX, 300 nM)-sensitive, membrane potential depolarization and [Ca^2+^]_i_ increase in endothelial cells. Interestingly, the endothelial cell depolarization was converted into a transient spike after removing Ca^2+^ ions from the buffer solution and in the presence of 100 *μ*M mibefradil or 10 *μ*M Ni^2+^. As expected, Ni^2+^ also abolished the increment in [Ca^2+^]_i_. Expression of Na_v_1.2, Na_v_1.6, and Ca_v_3.2 isoforms of voltage-dependent Na^+^ and Ca^2+^ channels was confirmed by immunocytochemistry. Furthermore, the Panx-1 channel blockade was associated with an increase in O_2_^·–^ production. Treatment with 10 *μ*M TEMPOL or 100 *μ*M apocynin prevented the increase in O_2_^·–^ formation, ACh-induced vasodilation, P-eNOS^S1177^, and P-Akt^S473^ observed in response to Panx-1 inhibition. These findings indicate that the Panx-1 channel blockade triggers a novel complex signaling pathway initiated by the sequential activation of TTX-sensitive Na_v_ channels and Ca_v_3.2 channels, leading to an increase in NO-mediated vasodilation through a NADPH oxidase-dependent P-eNOS^S1177^, which suggests that Panx-1 may be involved in the endothelium-dependent control of arterial blood pressure.

## 1. Introduction

Control of blood flow distribution relies on coordinated changes in the diameter of resistance arteries through a complex interplay between the vasoconstrictor and vasodilator signals that determines the degree of smooth muscle constriction (i.e., vasomotor tone). Endothelial cells play a critical role in this process by the activation of several signaling pathways that mediate the response initiated by different stimuli, which are, consequently, known as endothelium-dependent vasodilators [1, 2]. Nitric oxide (NO) has been recognized as the major vasodilator signal generated by endothelial cells; however, in small resistance arteries (i.e., feed arteries and arterioles), an additional vasodilator pathway associated with the NO-independent hyperpolarization of smooth muscle cells has also been described [2]. As this vasodilator pathway relies on the gap junction-mediated transmission to smooth muscle cells of a hyperpolarizing current initiated in the endothelium by the opening of Ca^2+^-activated K^+^ channels (K_Ca_) of small (SK_Ca_) and intermediate (IK_Ca_) conductance [2–4], this vasodilator component was termed as endothelium-derived hyperpolarization (EDH) [5]. In addition, to mediate the EDH signaling, gap junction communication has also been shown to provide a preferential signaling pathway for NO and contribute to the coordination of vasomotor tone in the microcirculation [6]. Although gap junctions play a central role in the coordination of vascular function, a functional association in endothelial cells between voltage-dependent Na^+^ channels (Na_v_) and T-type, voltage-dependent Ca^2+^ channels (Ca_v_3) of the subtype Ca_v_3.2 has also been proposed to participate in this process by supporting the conduction of vasodilator signals [2, 7, 8].

Gap junctions are intercellular channels formed by the serial docking of two hemichannels, each one provided by each neighboring cell, and, in turn, hemichannels are made up by the association of six protein subunits known as connexins. Interestingly, individual hemichannels might be functional, providing a pathway to connect the intra- and extracellular compartments [9–11]. Connexin-based channels may allow the release (hemichannels) or intercellular transfer (gap junction channels) of current, ions, and small signaling molecules (<1.4 nm diameter) such as ATP and IP_3_ [9–12]. In addition to connexins, channels formed by pannexins have emerged as an important signaling pathway for the control of vasomotor tone in resistance arteries [13]. Pannexins are a protein family structurally related to connexins; however, apparently, these proteins do not constitute functional gap junction channels in physiological conditions and only form membrane channels with similar characteristics to hemichannels, which have been proposed to function as a preferential pathway for ATP release [11, 14, 15].

Of the three pannexin isoforms described (Panx-1, Panx-2, and Panx-3), only Panx-1 has consistently been found in the vessel wall [16]. In resistance arteries, this pannexin isoform is expressed in endothelial and smooth muscle cells and the sympathetic nerve-triggered vasoconstriction initiated by *α*1-adrenoceptors has been reported to be mediated, in part, by ATP release from smooth muscle cells through Panx-1 channels [13, 16]. In contrast, in conduit arteries, Panx-1 is only expressed in the endothelium [16] and the activation of a Panx-1 channel-initiated purinergic signaling was found to contribute to the EDH-dependent vasodilation elicited by the endothelium-dependent vasodilator, acetylcholine (ACh) [17]. However, although the EDH-mediated vasodilator pathway was reduced in Panx-1 knockout mice, the NO-dependent vasodilator component was enhanced in these animals, suggesting that Panx-1 may be involved in the tonic regulation of NO production by the endothelial isoform of the enzyme endothelial NO synthase (eNOS) [17].

In the present study, we examined the effect of acute Panx-1 channel inhibition on the NO-dependent vasodilation induced by ACh in mesenteric resistance arteries. To this end, we evaluated the variations in vasomotor responses in relation to the changes in membrane potential and intracellular Ca^2+^ concentration ([Ca^2+^]_i_) observed in endothelial cells during the application of Panx-1 channel blockers. Our results show that the blockade of Panx-1 channels triggered a fast, transient, membrane depolarization-initiated increase in [Ca^2+^]_i_, which leads to the activation of a complex signaling cascade that evokes eNOS phosphorylation at serine 1177 (P-eNOS^S1177^) and the subsequent increment in the NO-mediated vasodilator component.

## 2. Materials and Methods

Male Sprague-Dawley rats (200-230 g) were bred and maintained in the Research Animal Facility of the Pontificia Universidad Católica de Chile. All experimental protocols were conducted according to the Helsinki Declaration and the Guiding Principles of Care and Use of Laboratory Animals endorsed by the American Physiological Society. In addition, the study was approved by the Institutional Bioethics Committee.

### 2.1. Perfusion of the Isolated Mesenteric Arterial Bed

Rats were anesthetized with xylazine and ketamine (10 and 90 mg/kg, i.p., respectively), and the mesenteric arterial bed was isolated as described by Lillo et al. [18]. Briefly, the superior mesenteric artery was cannulated and the mesentery was perfused at 2 mL/min with a warmed (37°C) Tyrode buffer solution (in mM: 118 NaCl, 5.4 KCl, 2.5 CaCl_2_, 1.2 KH_2_PO_4_, 1.2 MgSO_4_, 23.8 NaHCO_3_, and 11.1 glucose) that was bubbled with 95% O_2_-5% CO_2_ to yield pH 7.35-7.45. Immediately thereafter, the aorta was cut to ensure a fast killing by exsanguination and mesenteries were severed from the intestinal wall. Isolated mesenteric arterial beds were placed in a perfusion chamber, and the experiments were started after an equilibration period of 20 min. Changes in perfusion pressure were recorded by means of a pressure transducer (P23Db Statham) connected at the entrance of the superior mesenteric artery and the WinDaq software (DataQ Instruments Inc., USA). All drugs were applied dissolved in the perfusion solution.

### 2.2. Vasomotor Responses

In the isolated mesenteric preparation, the intraluminal pressure of resistance arteries is low and does not reach the threshold to activate a myogenic response. Therefore, vessels were constricted with 70 mM KCl, since the ACh-elicited vasodilation depends exclusively on NO in KCl-constricted mesenteries [6, 19]. High-KCl solutions were prepared by equimolar substitution of Na^+^ ions for K^+^ ions. The response to ACh was evaluated 15 min after blocking Panx-1 channels with 1 mM probenecid, and the results were expressed as a percentage of reduction in the perfusion pressure (% relaxation) or as a percentage of change in the perfusion pressure along the time (% baseline).

### 2.3. Primary Cultures of Mesenteric Endothelial Cells

Microvascular endothelial cells were isolated as described by Ashley et al. [20]. Briefly, after removing the blood from the vessels by perfusing a sterile Tyrode buffer solution containing a mixture of antibiotics and antimycotics (Anti-Anti solution, Gibco, Invitrogen, NY, USA), mesenteries were incubated in a physiological saline solution containing 0.2% collagenase type I and 0.1% BSA at 37°C. After 1 h, the collagenase/BSA solution was removed by two successive applications of M-199 media and centrifugation. Pelleted cells were resuspended in M-199 media containing 20 *μ*g/mL endothelial cell growth supplement from bovine pituitary (ECGS) and 20% fetal bovine serum (FBS) and seeded onto sterile glass coverslips. Nonadherent cells were removed 4 h later, and the remaining adherent endothelial cells were kept at 37°C in a 5% CO_2_-95% air atmosphere at nearly 100% relative humidity. To carry out the experiments, the culture media of endothelial cells of 70 to 80% of confluence (~2 days of culture) were replaced by a MOPS-buffered Tyrode saline solution (pH 7.4).

### 2.4. Measurements of Intracellular Ca^2+^ Concentration

Changes in [Ca^2+^]_i_ were detected using the fluorescent Ca^2+^ indicator, Fluo 4 (Life Technologies, OR, USA), as described previously [18]. Fluo 4 was uploaded by incubating the primary cultures of endothelial cells with 10 *μ*M Fluo 4-acetoxymethyl ester (Fluo 4-AM) for 1 h at room temperature (~25°C), and time-lapse measurements of [Ca^2+^]_i_ were started after 20 min of equilibration using an Olympus BX50 WI microscope and an intensified CCD camera (Retiga Fast 1394, QImaging) controlled by the IPLab software (Scanalytics, Inc.). Variations in fluorescence intensity were expressed as *F*/*F*_0_, where *F* is the fluorescence observed during the recording period and *F*_0_ is the baseline fluorescence value. Fluo 4-AM was prepared in DMSO and diluted to the working concentration in MOPS-buffered Tyrode solution.

### 2.5. Membrane Potential Recordings

Changes in membrane potential were recorded in primary cultures of endothelial cells and in smooth muscle cells of intact, isolated mesenteric resistance arteries (120–180 *μ*m inner diameter) using glass pulled microelectrodes filled with 3 M KCl (pipette resistance: 30–60 M*Ω*) connected to an electrometer DUO 773 (World Precision Instruments, Inc., FL, USA), as described by Lillo et al. [18]. In the case of smooth muscle recordings, resistance arteries were pinned down on a Sylgard® (Dow Corning Corporation, MI, USA) surface at the bottom of a chamber containing MOPS-buffered Tyrode solution (pH 7.4) and, to recognize the cell type impaled, the microelectrode filling solution also included 10 *μ*M dextran-FITC (MW: 3000 Da) in addition to 3 M KCl. The preparation was grounded with an Ag-AgCl reference electrode placed in the buffer solution, and, with the assistance of a microscope (Nikon Eclipse), the recording microelectrode was guided using an electronic micromanipulator (Burleigh TS-5000-I50, NY) to impale an endothelial cell or the isolated artery. Successful cell impalement was recognized by a rapid negative deflection of potential, stable membrane potential in basal conditions, and positive deflection on exit. Changes in membrane potential were recorded at 1000 Hz unless otherwise indicated, using the data acquisition software LabScribe (iWorx Systems, Inc., NH, USA).

### 2.6. Superoxide Anion Measurements

The superoxide anion (O_2_^·–^) probe dihydroethidine (DHE) was used to detect O_2_^·–^ formation in intact resistance arteries and in primary cultures of mesenteric endothelial cells [21]. To measure O_2_^·–^ in resistance arteries, isolated mesenteric arterial beds were perfused for 15 min with a Tyrode buffer solution containing 10 *μ*M DHE alone or in combination with Panx-1 channel blockers, probenecid (1 mM) or ^10^Panx (60 *μ*M). The effect of the vehicle of the blockers was also evaluated as a control. DHE and blockers were washed out for 10 min, and a small resistance artery (120–180 *μ*m inner diameter, ~1.0 cm length) was isolated and pinned down on a Sylgard® surface at the bottom of a 35 mm dish containing MOPS-buffered Tyrode solution (pH 7.4). A similar protocol was used in cultured endothelial cells, but, in this case, the time course of the DHE-generated signal was recorded. DHE diffuses into the cell and is oxidized by reactive oxygen species (ROS) to form ethidium, which produces nuclear fluorescence after intercalating with DNA. The fluorescent signal was examined by epifluorescence (exciter: 530-550 nm, band-pass filter; emission: 590 nm, long-pass filter) using an intensified CCD camera (Retiga Fast 1394, QImaging) and the IPLab software (Scanalytics, Inc.). The analysis of the fluorescence intensity was performed using the software ImageJ. As the DHE fluorescent signal is not specific for O_2_^·–^, these measurements were complemented with the direct O_2_^·–^ detection using the analysis by emitted light (ABEL®) assay, which is based on the intense luminescence emitted upon the reaction of O_2_^·–^ radicals with the prosthetic group of pholasin, the photoprotein responsible for luminescence in the bivalve *Pholas dactylus* [22, 23] (see Supplementary Materials (available here)).

### 2.7. Western Blot

Mesenteries were homogenized, and proteins were separated by 12% SDS-PAGE and transferred onto a PVDF membrane (Pierce, Rockford, IL, USA), as described previously [18]. The Signal Enhancer HIKARI (Nacalai Tesque, Inc., Japan) was used to incubate the primary (BD Transduction Labs, Lexington, KY, USA) and secondary antibodies (Pierce, Rockford, IL, USA), the molecular mass was estimated with prestained markers (Bio-Rad, Hercules, CA, USA), and the protein bands were detected with the SuperSignal® West Femto (Pierce, Rockford, IL, USA). Blots were developed for eNOS phosphorylation at serine 1177 (P-eNOS^S1177^) and then stripped three times successively to reprobe the membranes for total eNOS, Akt phosphorylation at serine 473 (P-Akt^S473^), and total Akt. Protein bands were analyzed using the ImageJ software, and changes in eNOS and Akt phosphorylation were expressed as the ratio of phosphorylated protein over total protein. In an additional experimental series, a group of mesenteries was perfused for 30 min with a Ca^2+^-free Tyrode buffer solution equilibrated with 95% O_2_-5% CO_2_ (pH 7.35-7.45) at 37°C as described above, but containing 0.5 mg/mL collagenase type II (Worthington, Lakewood, NJ, USA) to remove the endothelium. Then, mesenteric arteries were perfused with a control Tyrode buffer solution for an additional 5 min and the tissue was homogenized and prepared for Western blot analysis. In addition, expression of Na_v_1.2, Na_v_1.6, and Ca_v_3.2 channels in the plasma membrane was analyzed by biotinylation of cell surface proteins (see Supplementary Materials (available here)).

### 2.8. Immunocytochemistry Analysis

Mesenteric arterial beds were first perfused for 10 min and then incubated overnight with Bouin's solution to fix and postfix the tissue, respectively. Subsequently, mesenteric arteries were prepared for immunohistochemistry or immunofluorescence analysis, as described previously [7, 18]. Tissues were dehydrated, embedded in paraffin, sectioned (10 *μ*m), placed on charge-coated slides, and deparaffinized using standard procedures to analyze the expression of Na_v_1.2, Na_v_1.6, and Ca_v_3.2 channels by immunohistochemistry or Panx-1 and caveolin-1 (Cav-1) by immunofluorescence. The expression of Na_v_1.2, Na_v_1.6, and Ca_v_3.2 channels was also assessed by immunofluorescence in cultured endothelial cells fixed with 4% paraformaldehyde. For immunohistochemistry, tissue sections were blocked with 0.5% BSA in TBS (pH 7.4) for 1 h at room temperature and prepared as indicated by the Mouse/Rabbit ImmunoDetector System (Bio SB, Santa Barbara, CA, USA) protocol. After blocking the endogenous peroxidase activity, sections were incubated overnight at 4°C with anti-Na_v_1.2, anti-Na_v_1.6, or anti-Ca_v_3.2 rabbit primary antibodies (Alomone Laboratories, Israel) and the signal was developed using the biotin link secondary antibody (10 min), HPR label, and DAB chromogen of the Mouse/Rabbit ImmunoDetector System. For immunofluorescence, sections and endothelial cell monolayers were blocked with 0.5% BSA in PBS and incubated with the anti-Panx-1 rabbit primary antibody (Sigma-Aldrich, St. Louis, MO, USA) or anti-Cav-1 mouse primary antibody (BD Transduction Labs, Lexington, KY, USA) in the case of tissue sections or anti-Na_v_1.2, anti-Na_v_1.6, or anti-Ca_v_3.2 primary antibodies in the case of cultured cells and then with the appropriate Alexa Fluor 568-labeled goat anti-rabbit or anti-mouse secondary antibody (Molecular Probes, OR, USA) using the Signal Enhancer HIKARI (Nacalai Tesque, Inc., Japan) as indicated by the manufacturer. The fluorescent signal was examined using an Olympus BX41 WI microscope and a CCD camera (Jenoptik ProgRes C5).

### 2.9. Analysis of Panx-1 Subcellular Distribution

The potential subcellular localization of Panx-1 channels in caveolae was evaluated by assessing the spatial association between Panx-1 and Cav-1 using the Proximity Ligation Assay (PLA, Duolink II, Olink Bioscience, Sweden) as described previously [18]. Tissue sections (10 *μ*m) were blocked with 0.5% BSA and incubated with rabbit polyclonal anti-Panx-1 (Sigma-Aldrich) and mouse monoclonal anti-Cav-1 (BD Transduction Labs) primary antibodies, which were detected using oligonucleotide-conjugated secondary antibodies as described in the manufacturer's protocols. The oligonucleotides can meet each other if the proteins are closer than 20 nm and thus can be used as a template for DNA ligase-mediated joining of additional oligonucleotides to form a circular DNA molecule, which was amplified using hybridizing fluorophore-labeled oligonucleotides. Primary antibodies were omitted as a negative control. Images were visualized with an Olympus LSM FLUOVIEW 1000 confocal microscope.

### 2.10. Chemicals

All chemicals of analytical grade were obtained from Merck (Darmstadt, Germany). MOPS, EGTA, ECGS, BSA, DHE, ACh, probenecid, dextran-FITC (MW: 3000 Da), mibefradil, Ni^2+^, 18*β*-glycyrrhetinic acid (*β*-GA), methyl-*β*-cyclodextrin (M*β*CD), and N^G^-nitro-L-arginine (L-NA) were purchased from Sigma-Aldrich (St. Louis, MO, USA). Apocynin and TEMPOL were obtained from Calbiochem (La Jolla, CA, USA), tetrodotoxin (TTX) from Affix Scientific, and collagenase type I from Worthington (Lakewood, NJ, USA). The Panx-1 channel blocking peptide ^10^Panx was synthesized by GenScript (Israel). *β*-GA and apocynin were dissolved in DMSO and probenecid in 0.5 M NaOH. These inhibitors were then diluted in a buffer solution to reach the final working concentration (final amount of DMSO < 0.1% and 1.4 mM NaOH). DMSO did not have an effect per se (data not shown). The pH of the final solution of probenecid was checked before the experiments, and the effect of the vehicle was also evaluated.

### 2.11. Statistical Analysis

Values are represented as mean ± standard error. Comparisons between groups were made using paired or unpaired Student's *t*-test, one-way ANOVA plus the Newman-Keuls post hoc test, or two-way ANOVA as appropriate. *P* < 0.05 was considered significant.

## 3. Results

**Figure 1:**
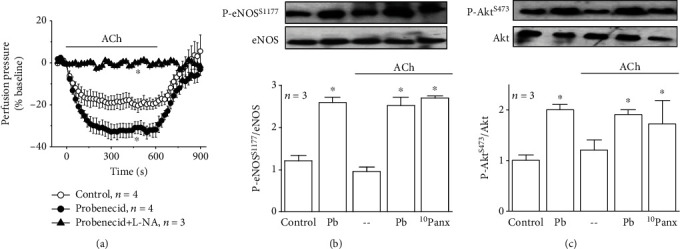
Blockade of Panx-1 channels enhances the NO-mediated vasodilation activated by ACh. (a) Time course of the vasodilation induced by 100 nM ACh in KCl-contracted arterial mesenteric beds in control conditions and in the presence (15 min) of 1 mM probenecid alone or in addition to the treatment for 45 min with 100 *μ*M N^G^-nitro-L-arginine (L-NA), a blocker of NO production. Probenecid was applied during the last 15 min of the treatment with L-NA. The horizontal bar indicates the period of stimulation. (b, c) Representative Western blots and densitometric analysis of eNOS (b) and Akt (c) expression as well as the phosphorylation of eNOS at serine 1177 (P-eNOS^S1177^, (b)) and Akt at serine 473 (P-Akt^S473^, (c)) observed in basal conditions (control) and after the treatment with 1 mM probenecid (Pb) or 60 *μ*M ^10^Panx. In the densitometric analysis, the changes in eNOS and Akt phosphorylation are expressed as the ratio of phosphorylated protein over total protein. Values are means ± SEM. ^∗^*P* < 0.05 vs. the control by one-way ANOVA plus the Newman-Keuls post hoc test.

The endothelium-dependent vasodilation was analyzed in mesenteric resistance arteries precontracted with 70 mM KCl. Perfusion pressure of mesenteric arterial beds was 3.7 ± 0.8 mmHg in resting conditions and increased during the stimulation with KCl to 20.9 ± 2.3 mmHg in 2-3 min (*n* = 6). Application of 100 nM ACh for 10 min evoked the relaxation of mesenteric resistance arteries, which was reflected in a rapid reduction in perfusion pressure that reached a maximum after ~2 min of stimulation and gradually returned to the KCl preconstriction level after the end of ACh application (Figure 1(a)).

### 3.1. Control of NO-Mediated Vasodilation by Panx-1 Channels

Mesenteric arteries were treated with probenecid to evaluate the participation of Panx-1-formed channels in response to ACh. Although probenecid application did not affect the basal perfusion pressure of mesenteric arterial beds (see Supplementary Fig. S1a and S1b), treatment with this blocker attenuated the vasoconstriction evoked by KCl (see Supplementary Fig. S2) and enhanced the ACh-induced vasodilation (Figure 1(a)). In KCl-contracted arteries, the endothelium-dependent vasodilation relies exclusively on NO production [6, 19] (see Supplementary Fig. S3) and, consistent with this notion, inhibition of NO production with 100 *μ*M L-NA abolished the vasodilation activated by ACh in the presence of probenecid (Figure 1(a)). Then, we evaluated the effect of the Panx-1 channel blockade on P-eNOS^S1177^, which is a regulatory mechanism that enhances Ca^2+^-mediated NO production. A basal P-eNOS^S1177^ was detected in control conditions, and the larger vasodilation observed in the presence of probenecid was associated with an increment in the P-eNOS^S1177^ level (Figure 1(b)) and also in Akt phosphorylation at serine 473 (P-Akt^S473^, Figure 1(c)), suggesting that the eNOS phosphorylation triggered by the Panx-1 channel blockade was mediated by the activation of the PI3K/Akt signaling pathway, as previously observed in response to different stimuli such as shear stress or bradykinin [24–26]. In line with previous reports [19], stimulation with ACh did not alter the level of P-eNOS^S1177^ and P-Akt^S473^ observed in basal conditions or after inhibiting Panx-1 channels with probenecid or the Panx-1 blocking peptide ^10^Panx (Figures 1(b) and 1(c)). Altogether, these results suggest that Panx-1 channels present a basal activity that may be involved in the regulation of endothelial cell function.

### 3.2. Voltage-Dependent Ca^2+^ Signaling Triggered by the Panx-1 Channel Blockade

**Figure 2:**
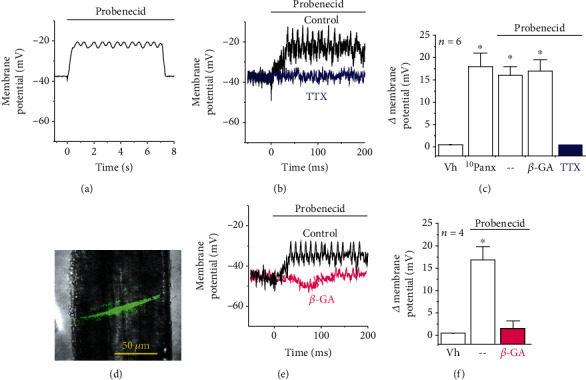
Blockade of Panx-1 triggers a rapid tetrodotoxin- (TTX-) sensitive depolarization of endothelial cell membrane potential. (a) Time course of the changes in membrane potential evoked by the Panx-1 channel blockade with 1 mM probenecid in primary cultures of endothelial cells. Membrane potential was recorded at 100 Hz. (b) Representative recordings of the membrane depolarization observed in primary cultures of endothelial cells in response to probenecid in control conditions and in the presence of 300 nM TTX. (c) Analysis of the maximum depolarization evoked by the application of 60 *μ*M ^10^Panx or 1 mM probenecid and its vehicle (Vh). Resting membrane potential was not affected by the vehicle of probenecid or ^10^Panx. The effect of 50 *μ*M 18*β*-glycyrrhetinic acid (*β*-GA) and TTX is also shown. (d) Representative image of a smooth muscle cell microinjected with dextran-FITC (3000 Da) during the recording of the changes in membrane potential from the smooth muscle layer of an isolated mesenteric resistance artery. (e) Representative recordings of the changes in membrane potential observed in intact arteries in response to the Panx-1 channel blockade with probenecid in control conditions and in the presence of *β*-GA. (f) Analysis of the maximum smooth muscle depolarization activated by probenecid in control conditions and in the presence of *β*-GA. The effect of the vehicle (Vh) of probenecid is also shown. Values are means ± SEM. ^∗^*P* < 0.05 vs. the vehicle by one-way ANOVA plus the Newman-Keuls post hoc test.

Control of membrane potential plays an important role in the regulation of endothelial cell signaling and in the Ca^2+^-dependent eNOS activation; thus, we evaluated the effect of the Panx-1 channel blockade on endothelial cell membrane potential. Surprisingly, in primary cultures of mesenteric endothelial cells, application of Panx-1 channel blockers, probenecid or ^10^Panx, evoked a fast tetrodotoxin- (TTX-) sensitive membrane depolarization that reached a maximum in ~40 ms and returned to the control level after a plateau phase of ~7 s (Figures 2(a)–2(c)). This response was also observed in smooth muscle cells of intact mesenteric resistance arteries (Figures 2(d)–2(f)). However, endothelial and smooth muscle cells are connected through gap junctions (i.e., myoendothelial gap junctions) and the inhibition of these intercellular channels with 18*β*-glycyrrhetinic acid (50 *μ*M, *β*-GA) completely prevented the smooth muscle cell depolarization elicited by probenecid (Figures 2(e) and 2(f)). In contrast, *β*-GA did not affect the response recorded in endothelial cells (Figure 2(c)), indicating that the depolarizing signal was triggered in the endothelium by the activation of TTX-sensitive, voltage-dependent Na^+^ channels (Na_v_) and thus transmitted to smooth muscle cells via myoendothelial gap junctions.

**Figure 3:**
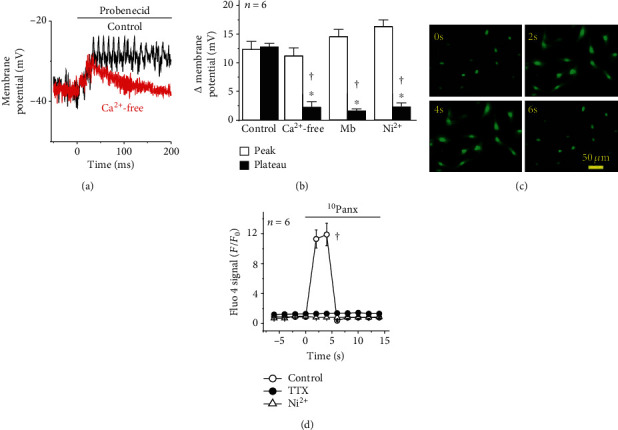
T-type voltage-dependent Ca^2+^ channels (T-type Ca_v_) are involved in the endothelial cell depolarization evoked by the Panx-1 channel blockade. (a) Representative recordings of the changes in endothelial cell membrane potential evoked by 1 mM probenecid in control conditions or after removing Ca^2+^ ions from the buffer solution (Ca^2+^-free solution plus 2 mM EGTA). Note that the absence of extracellular Ca^2+^ ions unmasked two components: an initial peak and a Ca^2+^-dependent plateau phase. Treatment with the Ca^2+^-free solution was initiated 5 min before probenecid application. (b) Analysis of the peak and plateau phase of the depolarization evoked by probenecid in control conditions and during the treatment with a Ca^2+^-free solution, 100 *μ*M mibefradil (Mb), or 10 *μ*M Ni^2+^. (c, d) Representative images (c) and quantitative analysis (d) of the changes in [Ca^2+^]_i_ observed in response to the Panx-1 channel blockade with 60 *μ*M ^10^Panx in primary cultures of endothelial cells. Note that tetrodotoxin (TTX, 300 nM) and Ni^2+^ abolished the increase in [Ca^2+^]_i_ activated by ^10^Panx. Values are means ± SEM. ^∗^*P* < 0.05 vs. the peak by paired Student's *t*-test. ^†^*P* < 0.05 vs. the control by one-way ANOVA plus the Newman-Keuls post hoc test.

The activation of Na_v_ channels is anticipated to be transient, which suggests the possible contribution of a second component in the endothelium-dependent depolarization triggered by the Panx-1 channel blockade, such as a Ca^2+^ influx. Consistent with this hypothesis, the plateau phase of the probenecid-activated depolarization was not observed after removing Ca^2+^ ions from the buffer solution (i.e., Ca^2+^-free solution) or in the presence of 100 *μ*M mibefradil or 10 *μ*M Ni^2+^ (Figures 3(a) and 3(b)), suggesting that the initial Na_v_-mediated depolarization was coupled to the subsequent activation of Ca_v_3 channels, most likely, the subtype Ca_v_3.2. In addition, the plateau of the depolarization was paralleled by a prominent increase in [Ca^2+^]_i_ (Figures 3(c) and 3(d)) that showed the same temporal characteristics of the change in membrane potential (Figure 2(a)) and, as expected, was abolished by Ni^2+^, but also by TTX (Figure 3(d)), supporting the notion that the activation of the Ca^2+^ signal was triggered by TTX-sensitive Na_v_ channels.

**Figure 4:**
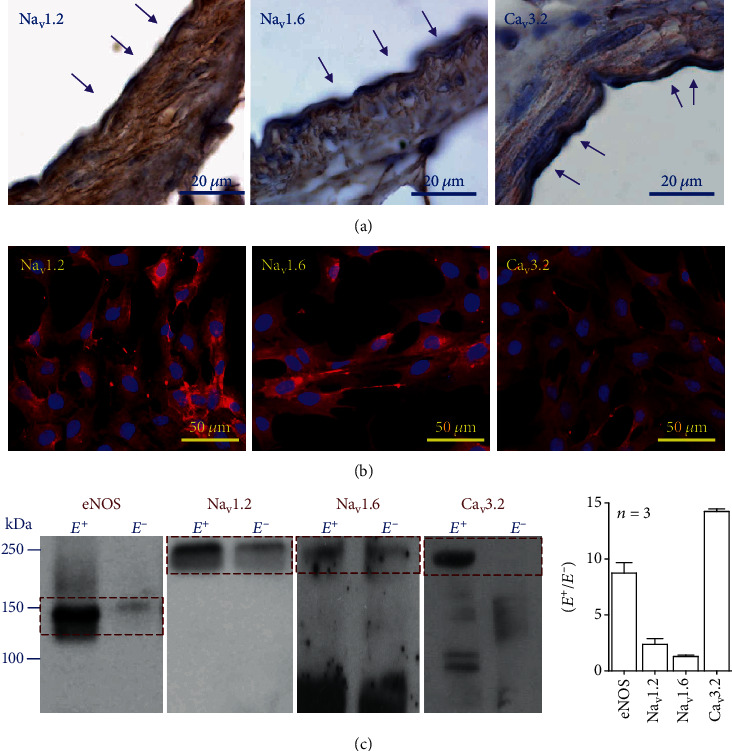
Expression of voltage-dependent Na^+^ (Na_v_) and Ca^2+^ (Ca_v_) channels in endothelial cells of mesenteric resistance arteries. (a) Immunohistochemistry analysis of the cellular distribution of Na_v_ and Ca_v_ channel-specific isoforms Na_v_1.2, Na_v_1.6, and Ca_v_3.2 in the wall of mesenteric resistance arteries. Note that Na_v_1.2 and Na_v_1.6 channels are present in both endothelial cells and smooth muscle cells, but Ca_v_3.2 channels are expressed exclusively in the endothelium. Arrows highlight the staining observed in endothelial cells. (b) Immunofluorescence detection of the expression of Na_v_1.2, Na_v_1.6, and Ca_v_3.2 channels in primary cultures of mesenteric endothelial cells. (c) Representative Western blots and densitometric analysis of the expression of eNOS and Na_v_1.2, Na_v_1.6, and Ca_v_3.2 channels in intact mesenteric arteries before (E^+^) and after (E^−^) removing the endothelium by the treatment with collagenase.

These findings suggest that TTX-sensitive Na_v_ channels and Ca_v_3.2 channels are present in endothelial cells and the expression of the isoforms Na_v_1.2 and Na_v_1.6 of TTX-sensitive Na_v_ channels as well as the isoform Ca_v_3.2 of Ca_v_3 channels has been detected in the endothelium [7, 27], which we confirmed by immunocytochemistry analysis in mesenteric resistance arteries. Both Na_v_1.2 and Na_v_1.6 channels were found to be expressed in endothelial cells as well as in smooth muscle cells, but, in contrast, the staining for Ca_v_3.2 channels was confined exclusively to the endothelium (Figure 4(a)). In addition, the expression of Na_v_1.2, Na_v_1.6, and Ca_v_3.2 channels in the endothelium was corroborated in primary cultures of mesenteric endothelial cells by immunofluorescence (Figure 4(b)) and in intact mesenteric resistance arteries with (E^+^) or without endothelium (E^−^) by Western blot (Figure 4(c)). Consistent with the immunocytochemistry analysis, removal of the endothelium by perfusing resistance vessels with collagenase resulted in a striking reduction in the Western blot signal for Na_v_1.2 and Na_v_1.6 channels, but the presence of Ca_v_3.2 channels was practically undetectable in endothelium-denuded vessels (Figure 4(c)), confirming the preferential endothelial cell expression of these channels in the wall of resistance arteries. In this experimental series, the reduction in the eNOS signal after endothelial cell removal was also evaluated as a control (Figure 4(c)). Na_v_1.2, Na_v_1.6, and Ca_v_3.2 channels must be found at the plasma membrane to be functional, which we further analyzed by biotinylation of surface proteins of the endothelial cell luminal membrane in intact mesenteric resistance vessels (see Supplementary Fig. S4). As expected, all three channels, Na_v_1.2, Na_v_1.6, and Ca_v_3.2, were found in the biotin-labeled protein fraction, in addition to the whole vessel sample (see Supplementary Fig. S4), indicating that these channels are expressed at the endothelial cell plasma membrane. It should be noted that perfusion of biotin did not reach intracellular proteins of endothelial cells or plasma membrane proteins of smooth muscle cells because this treatment did not target eNOS or L-type voltage-dependent Ca^2+^ channels, Ca_v_1.2, respectively. In contrast, a relevant membrane protein of endothelial cells, such as the Na^+^-Ca^2+^ exchanger, was also found in the biotin-labeled protein fraction (see Supplementary Fig. S4).

### 3.3. Panx-1 Subcellular Distribution in Resistance Arteries

**Figure 5:**
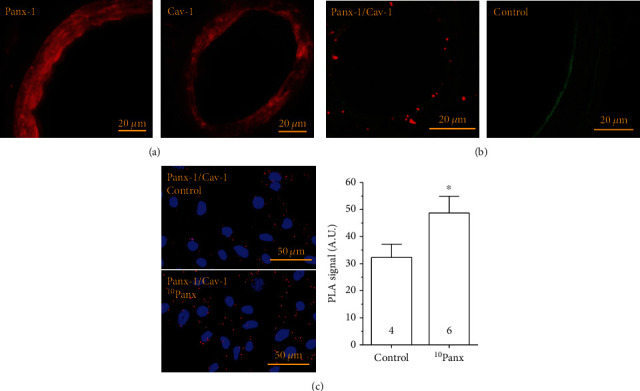
Panx-1 is associated with caveolin-1 (Cav-1) in endothelial cells. (a) Immunofluorescence analysis of the cellular distribution of Panx-1 (left) and Cav-1 (right) in mesenteric resistance arteries. (b) Analysis performed by Proximity Ligation Assay (PLA) of the spatial association of Panx-1 with Cav-1 (left) and the negative control (right) in which primary antibodies were omitted. Note that although Panx-1 and Cav-1 are expressed in endothelial cells and smooth muscle cells, the association between these two proteins is mainly observed in the endothelium. The green fluorescent signal corresponds to the internal elastic lamina. (c) Representative images (left) and fluorescence intensity analysis (right) of the PLA-detected association between Panx-1 and Cav-1 in primary cultures of mesenteric endothelial cells in control conditions and 5 min after the application of the Panx-1 blocking peptide ^10^Panx. Changes in the PLA signal are expressed in arbitrary units (A.U.). Numbers inside the bars indicate the *n* value. Values are means ± SEM. ^∗^*P* < 0.05 vs. the control by unpaired Student's *t*-test.

**Figure 6:**
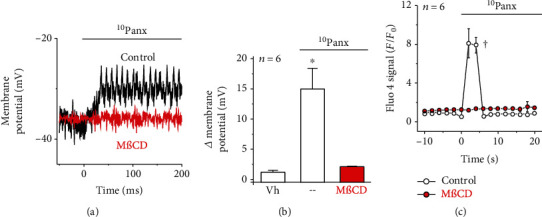
The depolarization and Ca^2+^ signaling activated in endothelial cells by the Panx-1 channel blockade depend on the integrity of cholesterol-rich microdomains. (a) Representative recordings of the changes in endothelial cell membrane potential evoked by 60 *μ*M ^10^Panx in control conditions and after disrupting the cholesterol-rich signaling microdomains by the treatment with 5 mM methyl-*β*-cyclodextrin (M*β*CD) for 30 min. (b) Analysis of the maximum depolarization evoked by ^10^Panx in control conditions and after the treatment with M*β*CD. (c) Time course of the changes in [Ca^2+^]_i_ observed in primary cultures of endothelial cells in response to ^10^Panx application before (control) and after the treatment with M*β*CD. Values are means ± SEM. ^∗^*P* < 0.05 vs. the vehicle by one-way ANOVA plus the Newman-Keuls post hoc test. ^†^*P* < 0.05 vs. the control by two-way ANOVA.

Signaling microdomains, such as caveolae, play a central role in the control of vascular function and in the regulation of NO production; therefore, we analyzed the cellular distribution of Panx-1 and Cav-1, a structural protein of caveolae, in mesenteric resistance arteries by immunofluorescence analysis and Proximity Ligation Assay (PLA). The fluorescent signal for Panx-1 and Cav-1 was detected in endothelial cells as well as in smooth muscle cells (Figure 5(a)), and the analysis of PLA revealed that both proteins are found in close spatial proximity mainly in endothelial cells (Figure 5(b)). The association of Panx-1 with Cav-1 was confirmed in primary cultures of mesenteric endothelial cells, and, interestingly, the blockade of Panx-1 channels with ^10^Panx evoked an increase in the level of spatial interaction between these two proteins (Figure 5(c)), which suggests that the organization of the signaling mechanism initiated by the Panx-1 channel blockade is orchestrated in caveolae. Consistent with this hypothesis, disruption of cholesterol-rich microdomains by treating the cultures of endothelial cells with 5 mM methyl-*β*-cyclodextrin (M*β*CD) for 30 min fully prevented the endothelial cell depolarization and the increase in [Ca^2+^]_i_ observed in response to ^10^Panx application (Figures 6(a)–6(c)).

### 3.4. Panx-1 Channel Blockade Leads to NADPH Oxidase-Derived Superoxide Formation

**Figure 7:**
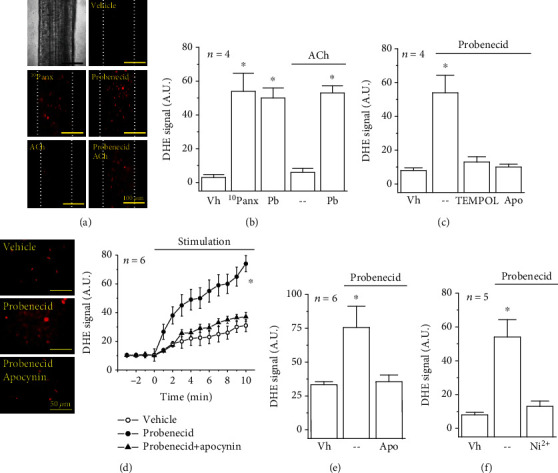
Blockade of Panx-1 channels leads to a NADPH oxidase-mediated increase in O_2_^·–^ formation. (a) Representative images of the O_2_^·–^-generated fluorescent signal observed in intact resistance arteries after 15 min application of 10 *μ*M dihydroethidine (DHE) alone (control, the vehicle (Vh) of probenecid) or with 60 *μ*M ^10^Panx, 1 mM probenecid (Pb), 100 nM ACh, or probenecid plus ACh. Dotted lines depict the outer edge of vessel walls. (b) Analysis of the O_2_^·–^ formation observed in the experiments shown in (a). (c) O_2_^·–^ production in response to probenecid in control conditions and in the presence of 10 *μ*M TEMPOL or 100 *μ*M apocynin (Apo). (d) Representative images and time course of the changes in the DHE signal observed in primary cultures of mesenteric endothelial cells in response to probenecid application in control conditions and in the presence of apocynin. The effect of the vehicle of probenecid is also shown. The horizontal bar indicates the period of stimulation. (e, f) Analysis of the maximum DHE signal attained in endothelial cell cultures in response to probenecid in control conditions and during the treatment with apocynin (e) or Ni^2+^ (f). Changes in the DHE-derived fluorescent signal are expressed in arbitrary units (A.U.). Values are means ± SEM. ^∗^*P* < 0.05 vs. the vehicle by one-way ANOVA plus the Newman-Keuls post hoc test.

As depolarization of membrane potential as well as an increase in [Ca^2+^]_i_ may trigger the activation of NADPH oxidase in endothelial cells [28–30], we used DHE to assess O_2_^·–^ production in intact mesenteric resistance arteries and primary cultures of mesenteric endothelial cells. The blockade of Panx-1 channels with probenecid or ^10^Panx in resistance vessels resulted in a strong increment in the DHE-generated fluorescent signal (Figures 7(a) and 7(b)) that was fully prevented by 10 *μ*M TEMPOL (Figure 7(c)), a O_2_^·–^ dismutase mimetic, confirming that the increase in the DHE signal reflected O_2_^·–^ production. Additionally, the rise in O_2_^·–^ levels observed in response to the Panx-1 channel blockade was also corroborated using the photoprotein pholasin (see Supplementary Fig. S5). As expected, stimulation with ACh did not change the O_2_^·–^ levels observed in control conditions or after the treatment with probenecid (Figures 7(a) and 7(b)). The activation of O_2_^·–^ formation was rapid, since, in cultured endothelial cells, the increase in the DHE signal started immediately after probenecid application (Figure 7(d)). In agreement with the participation of NADPH oxidase in the response, the increase in O_2_^·–^ was abolished by 100 *μ*M apocynin, an inhibitor of NADPH oxidase, in both resistance arteries (Figure 7(c)) and endothelial cell cultures (Figures 7(d) and 7(e)). In addition to TEMPOL and apocynin, O_2_^·–^ formation was also inhibited by 10 *μ*M Ni^2+^ (Figure 7(f)), supporting the involvement of Ca_v_3.2 channels in the NADPH oxidase activation.

### 3.5. NADPH Oxidase Mediates the eNOS Activation Triggered by the Panx-1 Channel Blockade

**Figure 8:**
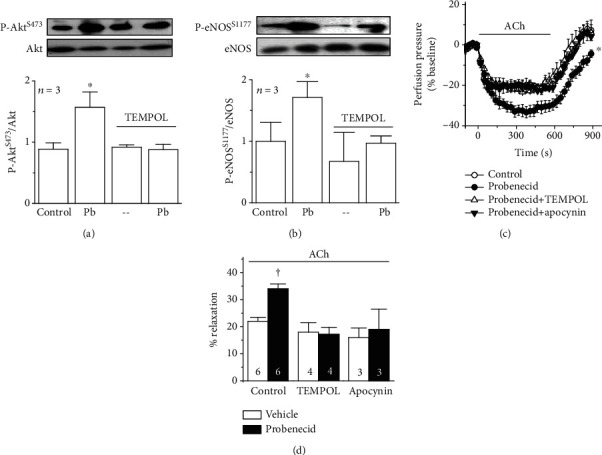
The activation of Akt signaling and eNOS phosphorylation initiated by the Panx-1 channel blockade depends on NADPH oxidase-mediated O_2_^·–^ production. (a, b) Representative Western blots and densitometric analysis of Akt (a), eNOS (b), Akt phosphorylation at serine 473 (P-Akt^S473^, (a)), and eNOS phosphorylation at serine 1177 (P-eNOS^S1177^, (b)) observed in basal conditions (control) and during the treatment with 1 mM probenecid (Pb) in the absence or presence of 10 *μ*M TEMPOL. The effect of TEMPOL alone is also shown. In the densitometric analysis, the changes in eNOS and Akt phosphorylation are expressed as the ratio of phosphorylated protein over total protein. (c) Time course of the vasodilation induced by 100 nM ACh in control conditions and in the presence of 1 mM probenecid alone or in combination with 10 *μ*M TEMPOL or 100 *μ*M apocynin. Arterial mesenteric beds were constricted with 70 mM KCl. The horizontal bar indicates the period of stimulation. (d) Maximum ACh-induced vasodilation attained during the application of probenecid or its vehicle in control conditions and in the presence of TEMPOL or apocynin. Numbers inside the bars indicate the *n* value. Values are means ± SEM. ^∗^*P* < 0.05 vs. the control by one-way ANOVA plus the Newman-Keuls post hoc test. ^†^*P* < 0.05 vs the vehicle by paired Student's t-test.

The NADPH oxidase-derived O_2_^·–^ production initiated by the Panx-1 channel blockade may lead to the activation of Akt-mediated signaling [31]; therefore, we evaluated if the NADPH oxidase/O_2_^·–^/Akt pathway was involved in the P-eNOS^S1177^-associated increase in NO-dependent vasodilation. Consistent with this hypothesis, the increase in the level of P-Akt^S473^ and P-eNOS^S1177^ observed after probenecid application was not evident in the presence of 10 *μ*M TEMPOL (Figures 8(a) and 8(b)). In line with these results, treatment with TEMPOL or apocynin (100 *μ*M) completely inhibited both the increment in the ACh-induced vasodilation (Figures 8(c) and 8(d)) and the reduction in the KCl-evoked vasoconstriction (see Supplementary Fig. S6a and S6b) attained after blocking Panx-1 channels with probenecid, which strongly support the involvement of the NADPH oxidase/O_2_^·–^ pathway in the Panx-1-mediated regulation of NO-dependent vasodilation.

## 4. Discussion

The relevance of Panx-1 channels in the control of endothelium-mediated vasomotor signaling is not clear. In spite of this cavity in the Panx physiology, deletion of Panx-1 was reported to attenuate the EDH pathway, and, interestingly, a compensatory increase in the NO-mediated vasodilation was also observed in the absence of this protein, suggesting that EDH or directly Panx-1 channels may be involved in a negative feedback mechanism that restrains NO production [17]. However, in contrast to this notion, our results show that the acute blockade of Panx-1 channels triggers a complex signaling pathway that leads to P-eNOS^S1177^ in response to a NADPH oxidase-mediated increase in O_2_^·–^ formation. Interestingly, this mechanism is triggered by a TTX-sensitive depolarization that is coupled to a Ca^2+^ influx through Ca_v_3 channels, apparently, the subtype Ca_v_3.2.

Blood flow distribution is controlled by the fine regulation of the diameter of small resistance arteries. In these arteries, Panx-1 is expressed not only in endothelial cells but also in smooth muscle cells [16] (Figure 5). Although the physiological relevance of Panx-1-mediated signaling in the control of vascular function is just beginning to be understood, channels formed by Panx-1 have been shown to be involved in the vasoconstriction initiated by the stimulation of *α*1-adrenoceptors in smooth muscle cells of resistance arteries [13] and in the EDH-mediated vasodilator component activated by ACh in conduit arteries [17]. Furthermore, Panx-1 has also been associated with the regulation of NO signaling, but the mechanism involved in this process has not been determined [17]. Therefore, to focus on the NO-dependent vasodilator component elicited by ACh, without interfering with the Panx-1-mediated vasoconstrictor signaling initiated by *α*1-adrenoceptors in smooth muscle cells, we used KCl-contracted resistance arteries to disable the EDH signaling and evoke a receptor-independent contraction. The ACh-induced vasodilation depends exclusively on NO in these conditions [19, 32] (Supplementary Fig. S3), and, therefore, the increase in response to ACh observed after the treatment with probenecid (Figure 1) confirmed that the blockade of Panx-1 channels enhances the endothelium-mediated NO signaling, as further demonstrated by the inhibition of NO production with L-NA (Figure 1). Consistent with increased NO production, probenecid also attenuated the KCl-evoked vasoconstriction (see Supplementary Fig. S2). Interestingly, the increase in the response was associated with an increment in P-eNOS^S1177^ (Figure 1), which is consistent with the upregulation of the relaxation, since eNOS is a Ca^2+^-dependent enzyme and P-eNOS^S1177^ is a well-characterized regulatory mechanism that enhances Ca^2+^-activated NO production [33, 34]. Activation of the PI3K/Akt signaling pathway leads to P-eNOS^S1177^ [24, 25, 35], and, in line with the participation of this pathway in the phosphorylation of eNOS, the Panx-1 channel blockade was also coupled to an increase in P-Akt^S473^ (Figure 1).

NO production and eNOS activity have been reported to be modulated by changes in endothelial cell membrane potential, and Panx-1 may work as a Cl^−^-selective channel in basal conditions [36], which may contribute to depolarize the endothelial cell membrane potential [37]. Then, we hypothesized that disruption of Panx-1 channel function may lead to an increase in NO production by triggering the hyperpolarization of membrane potential. Unexpectedly, the application of probenecid or ^10^Panx evoked a rapid membrane potential depolarization in primary cultures of mesenteric endothelial cells as well as in smooth muscle cells of intact mesenteric resistance arteries (Figure 2). In resistance arteries, endothelial and smooth muscle cells are communicated through myoendothelial gap junctions [2, 38] and inhibition of connexin-formed channels with *β*-GA prevented the smooth muscle depolarization but did not affect the response in endothelial cells (Figure 2), confirming that the depolarizing signal was triggered in the endothelium and was subsequently transmitted to smooth muscle cells via myoendothelial gap junctions. Therefore, these results suggest that, in endothelial cells, Panx-1 channels may be coupled to the regulation of a depolarizing mechanism, such as Na_v_ channels. Although voltage-dependent channels are not generally thought to be present in the endothelium, functional expression of Na_v_ channels has been detected in endothelial cells [7, 39–41]. In this context, Na_v_ channels have been reported to be involved in the endothelial response to shear stress [41] and to mediate the endothelium-dependent conducted vasodilation activated by depolarizing electrical stimulation of mouse cremaster arterioles [7]. Consistent with the functional association of these channels with Panx-1, the change in membrane potential triggered by the Panx-1 channel blockade was fully prevented by TTX (Figure 2). As expected, the activation of Na_v_ channels was transient (i.e., ~40 ms) and only contributed to the uprising phase of the depolarization, but, in addition, this initial phase triggered a Ca^2+^ influx that accounted for the sustained phase of the response, as demonstrated by the removal of Ca^2+^ ions from the bathing solution or the treatment with mibefradil or low concentrations of Ni^2+^ (10 *μ*M). In agreement with these results, application of the Panx-1 blocking peptide ^10^Panx elicited a rapid, strong increase in [Ca^2+^]_i_ that paralleled the depolarization and was sensitive to TTX and Ni^2+^ (Figure 3). Mibefradil is a blocker of Ca_v_3 channels, and Ni^2+^, at low concentrations such as 10 *μ*M, is a preferential inhibitor of the subtype Ca_v_3.2 of Ca_v_3 channels [42, 43]. Interestingly, in the wall of resistance arteries, these channels are expressed exclusively in the endothelium [8, 27] and were shown to be essential for normal relaxation of the murine coronary arteries [44]. Furthermore, our results confirmed the expression of Na_v_1.2, Na_v_1.6, and Ca_v_3.2 channels in endothelial cells of rat mesenteric resistance arteries (Figure 4 and Supplementary Fig. S4). Taken together, these data indicate that the blockade of Panx-1 channels leads to the activation of a complex mechanism based on the functional coupling between TTX-sensitive Na_v_ channels and Ca_v_3.2 channels, as that previously proposed to mediate the electrically induced endothelium-dependent conducted vasodilation in mouse cremaster arterioles [2, 7].

The mechanism by which the blockade of Panx-1 channels triggers the activation of Na_v_ channels requires further investigation. However, it is interesting that although Panx-1 is expressed in both endothelial cells and smooth muscle cells of mesenteric resistance arteries (Figure 5), the analysis of protein association by PLA revealed that Panx-1 is found in close spatial relation with Cav-1 mainly in the endothelium (Figure 5). Cav-1 is a structural protein of caveolae [45], and the special location of Panx-1 in these signaling microdomains may provide the functional organization required to trigger the coordinated activation of Na_v_ and Ca_v_3.2 channels by Panx-1 channel blockers, as these channels have been detected to be expressed in caveolae [46–48]. Interestingly, the application of Panx-1 channel blockers evokes an increase in the spatial association of Panx-1 with Cav-1 (Figure 5), which further supports the involvement of these microdomains in the response activated by the blockers. Consistent with this notion, disruption of cholesterol-rich microdomains, such as caveolae, by the treatment with M*β*CD [49] fully prevented the ^10^Panx-activated depolarization and [Ca^2+^]_i_ increase in cultured endothelial cells (Figure 6). These results indicate that caveolae play a central role in the signaling mechanism triggered by Panx-1 channel blockers and suggest that the analysis of protein-protein direct molecular interactions may help us to elucidate the functional connection between the Panx-1 and Na_v_ channels, but the activation of a Panx-1-mediated receptor-like signaling cascade by probenecid or ^10^Panx cannot be ruled out.

In endothelial cells, both depolarization of membrane potential and an increase in [Ca^2+^]_i_ have been shown to lead to a rise in O_2_^·–^ production by the activation of NADPH oxidase [28–30, 50, 51]. In addition, an increment in O_2_^·–^ may lead to the activation of the PI3K/Akt signaling pathway and, thereby, to the further increase in P-eNOS^S1177^ [31, 52]. Three NADPH oxidase isoforms are expressed in the endothelium of rats: NOX1 oxidase, NOX2 oxidase, and NOX4 oxidase. Interestingly, of these three isoforms, only NOX1 and NOX2 generate O_2_^·–^, since NOX4 mainly releases hydrogen peroxide (H_2_O_2_) [31, 53, 54]. Although the DHE-based analysis used in this study does not distinguish between O_2_^·–^ and H_2_O_2_, these measurements were complemented with the direct O_2_^·–^ detection by emitted light (ABEL®) assay (Supplementary Fig. S5), which supports the participation of NOX1- or NOX2-mediated O_2_^·–^ formation in the response activated by the Panx-1 channel blockade. Consistent with this notion, the increase in the DHE signal was fully blocked by both apocynin and TEMPOL (Figure 7). In this context, it should be noted that the effect of apocynin relies on the inhibition of the p47phox subunit association with the membrane-bound heterodimer of the NADPH oxidase complex (NOX and p22phox), which is a critical process for NOX1 and NOX2 activation, but not for NOX4 function [53]. Likewise, TEMPOL is a superoxide dismutase mimetic, and thus, it buffers the increase in O_2_^·–^ with the consequent H_2_O_2_ production [55]. Altogether, these data indicate that the response triggered by Panx-1 blockers was associated with an increase in O_2_^·–^ formation in endothelial cells of resistance arteries (Figure 7), although the involvement of NOX1 or NOX2 in this process must be confirmed in future investigations. Furthermore, the treatment with apocynin or TEMPOL prevented the increase in P-Akt^S473^ and P-eNOS^S1177^ and the increment in the magnitude of the ACh-induced vasodilation (Figure 8), confirming the participation of the NADPH oxidase/O_2_^·–^ in the Panx-1-mediated control of vasomotor tone by the regulation of NO production.

It is interesting to note that Panx-1-mediated signaling may be involved in the endothelium-dependent control of peripheral vascular resistance and, consequently, arterial blood pressure. In this context, probenecid treatment *in vivo* has been shown to elicit a reduction in systolic blood pressure in spontaneously hypertensive rats and an increment in leg vascular conductance in humans [56, 57]. As ATP release through Panx-1 channels has been reported to contribute to the vasoconstrictor response initiated by *α*1-adrenoceptors in smooth muscle cells of resistance arteries [13], the effects of Panx-1 blockers on peripheral vascular resistance have mostly been attributed to the disruption of the purinergic-mediated component of sympathetic nerve-triggered *α*1-adrenoceptor activation, although a possible direct interaction of probenecid with *α*-adrenoceptors has been also proposed [56]. However, in view of our results, an increase in the NO-mediated vasodilator component through an increment in P-eNOS^S1177^ may also be involved in the hypotensive response evoked by Panx-1 channel blockers, which highlights the potential relevance of endothelial cell Panx-1 in the control of vascular function.

## 5. Conclusions

Panx-1 channels have typically been described as a transmembrane pathway for ATP release, and accordingly, acute inhibition of the channel function is associated with the disruption of purinergic signaling [58]. However, at least in endothelial cells of resistance arteries, Panx-1 signaling seems to be more complex and is linked to eNOS phosphorylation through a NADPH oxidase/O_2_^·–^-mediated pathway. The results of the present work indicate that the blockade of Panx-1 channels leads to the activation of TTX-sensitive Na_v_ channels and the parallel recruitment of Panx-1 into caveolae, in association with Cav-1. The Na_v_ channel-mediated depolarizing current is coupled to the Ca_v_3.2 opening and the subsequent Ca^2+^ entry. Apparently, caveolae provide a signaling platform for the functional association between the Panx-1 and Na_v_ channels. The concomitant endothelial cell depolarization and [Ca^2+^]_i_ increase elicit the further activation of the NADPH oxidase/O_2_^·–^ signaling, which triggers the PI3K/Akt pathway and the subsequent increase in the ACh-induced NO-mediated vasodilation through the modulation of eNOS activity by the phosphorylation of the enzyme at serine 1177. These findings are thus consistent with the discovery of a novel regulation mechanism of NO production and highlight the relevance of Panx-1 and Na_v_ and Ca_v_3 channels in the control of endothelial cell function. This Panx-1-dependent signaling pathway is likely to play a critical role in the tonic, endothelial control of arterial blood pressure and, therefore, may contribute to the design of new therapeutic strategies for the treatment of cardiovascular-related diseases such as hypertension.

## Data Availability

The data used to support the findings of this study are available from the corresponding author upon request.

## Supplementary Materials

Supplementary Materials Supplementary Figure S1. Pannexin-1 channel blockade with probenecid (Pb) does not affect the basal perfusion pressure of mesenteric arterial beds. (a) Time course of the perfusion pressure in mesenteric arterial beds before and after probenecid application. (b) Level of perfusion pressure observed in mesenteric arteries during 10 min in control conditions or in the presence of probenecid. The horizontal bar indicates the period of probenecid application. Values are means ± SEM. Supplementary Figure S2. Inhibition of the KCl-evoked vasoconstriction by pannexin-1 channel blockade with probenecid (Pb). (a) and (b) Time course (a) and maximum increase in perfusion pressure (b) elicited by KCl (70 mM) in mesenteric arteries in control conditions and in the presence of probenecid (15 min). The horizontal bar indicates the period of KCl stimulation. Values are means ± SEM. ∗, *P* < 0.05 vs Control by paired Student's t test. Supplementary Figure S3. The vasodilation induced by acetylcholine (ACh) depends exclusively on NO production in KCl-contracted resistance arteries. Time course of changes in perfusion pressure elicited by 1 min stimulation with 100 nM ACh in control conditions and after blocking NO production with 100 *μ*M N*^G^*-nitro-L-arginine (L-NA) in arterial mesenteric vessels contracted with 70 mM KCl. The horizontal bar indicates the period of ACh stimulation. Values are means ± SEM. Supplementary Figure S4. Plasma membrane expression of Na_v_1.2, Na_v_1.6 and Ca_v_3.2 channels in mesenteric resistance arteries. The presence of these channels in the plasma membrane was analyzed by biotinylation of cell surface proteins. Mesenteric arteries were perfused with 1 mM sulfosuccinimidyl-2-[biotinamido]ethyl-1,3-dithiopropionate for 40 min at 0.5 mL/min to label membrane proteins of the luminal surface of the vessels. Then, tissues were homogenized and proteins were precipitated with ice-cold acetone. The pellet was suspended in Tris-buffer, the biotinylated proteins were captured with streptavidin–agarose beads and separated by SDS-PAGE to be detected with specific antibodies. A representative Western blot of three independent experiments is shown. The presence of eNOS, Na^+^-Ca^2^^+^ exchanger and the channels Na_v_1.2, Na_v_1.6, Ca_v_3.2 and Ca_v_1.2 was evaluated in the total tissue homogenized (**H**) and in the fraction of biotinylated-plasma membrane proteins (**M**). In addition to Na_v_1.2, Na_v_1.6 and Ca_v_3.2 channels, eNOS and Ca_v_1.2 were also analyzed as negative control to confirm that the biotin treatment did not label intracellular proteins of endothelial cells or plasma membrane proteins of smooth muscle cells. In addition, Na^+^-Ca^2+^ exchanger (NCX) was used as a positive control for the detection of plasma membrane proteins of endothelial cells. Supplementary Figure S5. Superoxide anion (O_2_^.–^) production activated by probenecid (Pb). O_2_^.–^ levels were measured in isolated mesenteric arterial beds of rats using the analysis by emitted light (ABEL®) assay, which is based on the intense luminescence emitted upon reaction of O_2_^.–^ radicals with the prosthetic group of Pholasin, the photoprotein responsible for luminescence in the bivalve Pholas dactylus. After treating mesenteric vessels with the pannexin-1 channel blocker, probenecid, or its vehicle, 20 *μ*L of perfusate samples were mixed with 100 *μ*L of adjuvant-K™ solution and, following 1 min of equilibration, the reaction was initiated by the injection of 250 *μ*L of Pholasin solution (10 *μ*g/mL). The resulting luminescence was immediately measured on a Turner TD20e luminometer (Promega). Adjuvant-K™ and pholasin solutions were prepared using the reconstitution buffer (Hank's balanced salt solution with 20 mM HEPES, pH 7.4) as indicated by the manufacturer (Knight Scientific Ltd.). Changes in O_2_^.–^ formation are expressed in arbitrary units (AU). Numbers inside the bars indicate the n value. All measurements were made in duplicate. Values are means ± SEM. ∗, *P* < 0.05 vs Vehicle by unpaired Student's t test. Supplementary Figure S6. The inhibition of the KCl-elicited vasoconstriction evoked by pannexin-1 channel blockade with probenecid (Pb) depends on a NADPH oxidase-initiated superoxide anion signaling. (a) and (b) Effect of probenecid on the time course (Left) and maximum increase in perfusion pressure (Right) evoked by KCl (70 mM) in mesenteric arteries treated with TEMPOL (a), a superoxide scavenger, or apocynin (b), a NADPH oxidase inhibitor. Note that the reduction in the KCl-induced vasoconstriction observed after pannexin-1 channel blockade with probenecid (Supplementary Fig. S2) is fully prevented by TEMPOL. (a) and apocynin (b). Horizontal bars indicate the period of KCl stimulation (Left) or the treatment applied (Right). Values are means ± SEM.
