# Supplementary material for: Novel Pannexin-1-Coupled Signaling Cascade Involved in the Control of Endothelial Cell Function and NO-Dependent Relaxation
Source: Oxid Med Cell Longev. 2021 Feb 20;2021:2678134. doi: 10.1155/2021/2678134 (PMC7914086; doi:10.1155/2021/2678134)
Supplement: Supplementary Materials — Supplementary Figure S1. Pannexin-1 channel blockade with probenecid (Pb) does not affect the basal perfusion pressure of mesenteric arterial beds. (a) Time course of the perfusion pressure in mesenteric arterial beds before and after probenecid application. (b) Level of perfusion pressure observed in mesenteric arteries during 10 min in control conditions or in the presence of probenecid. The horizontal bar indicates the period of probenecid application. Values are means ± SEM. Supplementary Figure S2. Inhibition of the KCl-evoked vasoconstriction by pannexin-1 channel blockade with probenecid (Pb). (a) and (b) Time course (a) and maximum increase in perfusion pressure (b) elicited by KCl (70 mM) in mesenteric arteries in control conditions and in the presence of probenecid (15 min). The horizontal bar indicates the period of KCl stimulation. Values are means ± SEM. ∗, P < 0.05 vs Control by paired Student's t test. Supplementary Figure S3. The vasodilation induced by acetylcholine (ACh) depends exclusively on NO production in KCl-contracted resistance arteries. Time course of changes in perfusion pressure elicited by 1 min stimulation with 100 nM ACh in control conditions and after blocking NO production with 100 μM NG-nitro-L-arginine (L-NA) in arterial mesenteric vessels contracted with 70 mM KCl. The horizontal bar indicates the period of ACh stimulation. Values are means ± SEM. Supplementary Figure S4. Plasma membrane expression of Nav1.2, Nav1.6 and Cav3.2 channels in mesenteric resistance arteries. The presence of these channels in the plasma membrane was analyzed by biotinylation of cell surface proteins. Mesenteric arteries were perfused with 1 mM sulfosuccinimidyl-2-[biotinamido]ethyl-1,3-dithiopropionate for 40 min at 0.5 mL/min to label membrane proteins of the luminal surface of the vessels. Then, tissues were homogenized and proteins were precipitated with ice-cold acetone. The pellet was suspended in Tris-buffer, the biotinylated proteins were captur [file 2678134.f1.pdf]

SUPPLEMENTARY MATERIAL

Novel pannexin-1-coupled signaling cascade involved in the control of endothelial cell function and NO-dependent relaxation

Mauricio A. Lillo[1]; Pablo S. Gaete[1]; Mariela Puebla[2]; Pía C. Burboa[1]; Inés Poblete[1]; Xavier F. Figueroa[1*]

[1]Departamento de Fisiología, Facultad de Ciencias Biológicas, Pontificia Universidad Católica de Chile, Santiago 8330025, Chile.

[2]Centro de Fisiología Celular e Integrativa, Facultad de Medicina-Clínica Alemana, Universidad del Desarrollo, Santiago, Chile.

*** Correspondence should be addressed to:**

Dr. Xavier F. Figueroa

Departamento de Fisiología

Facultad de Ciencias Biológicas

Pontificia Universidad Católica de Chile

Santiago, Chile

Phone: 562-2686-2356

E-mail: xfigueroa@bio.puc.cl

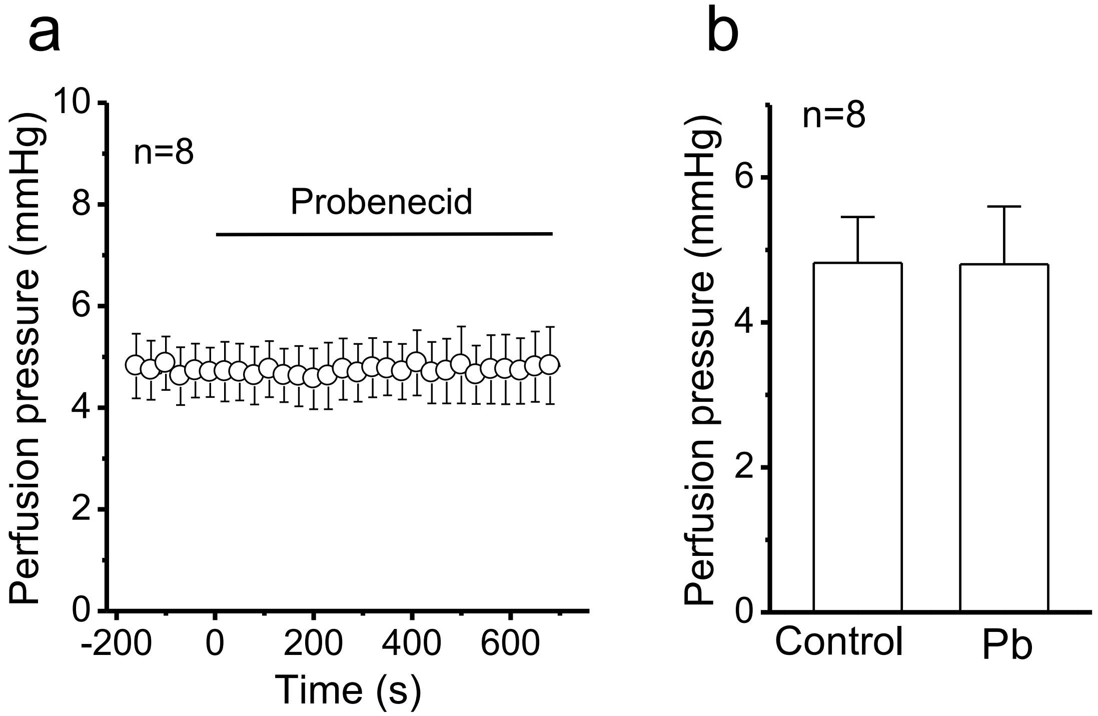

Supplementary Figure S1. Pannexin-1 channel blockade with probenecid (Pb) does not affect the basal perfusion pressure of mesenteric arterial beds. **a**, Time course of the perfusion pressure in mesenteric arterial beds before and after probenecid application. **b,** Level of perfusion pressure observed in mesenteric arteries during 10 min in control conditions or in the presence of probenecid. The horizontal bar indicates the period of probenecid application. Values are means ± SEM.

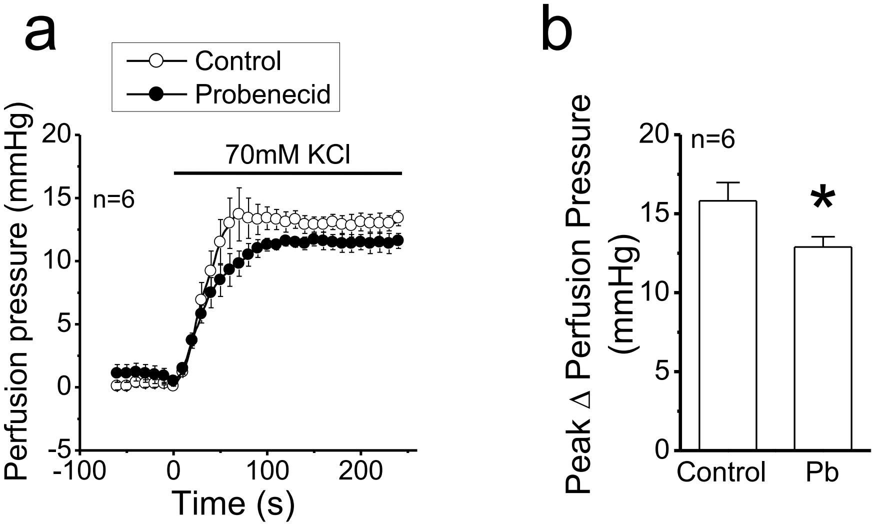

Supplementary Figure S2. Inhibition of the KCl-evoked vasoconstriction by pannexin-1 channel blockade with probenecid (Pb). **a** and **b**, Time course (**a**) and maximum increase in perfusion pressure (**b**) elicited by KCl (70 mM) in mesenteric arteries in control conditions and in the presence of probenecid (15 min). The horizontal bar indicates the period of KCl stimulation. Values are means ± SEM. *, $P<0.05$ vs Control by paired Student's t test.

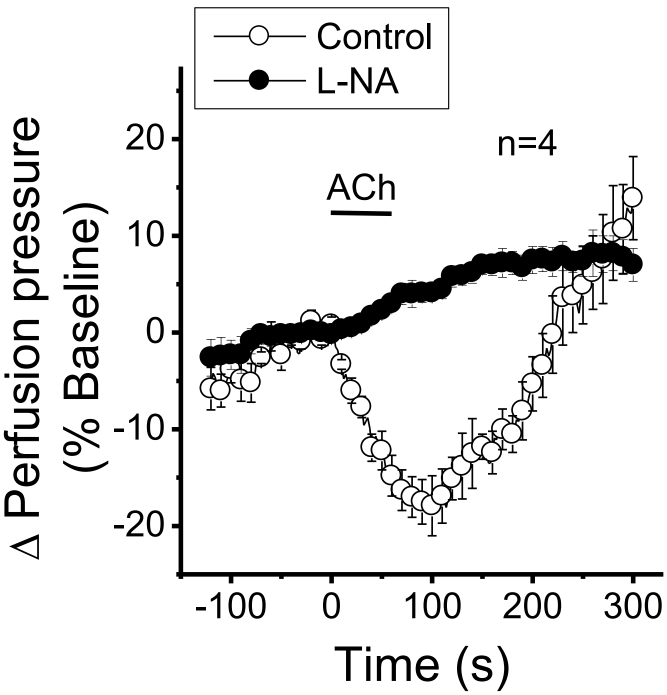

Supplementary Figure S3. The vasodilation induced by acetylcholine (ACh) depends exclusively on NO production in KCl-contracted resistance arteries. Time course of changes in perfusion pressure elicited by 1 min stimulation with 100 nM ACh in control conditions and after blocking NO production with 100 µM N^G-nitro-L-arginine (L-NA) in arterial mesenteric vessels contracted with 70 mM KCl. The horizontal bar indicates the period of ACh stimulation. Values are means ± SEM.

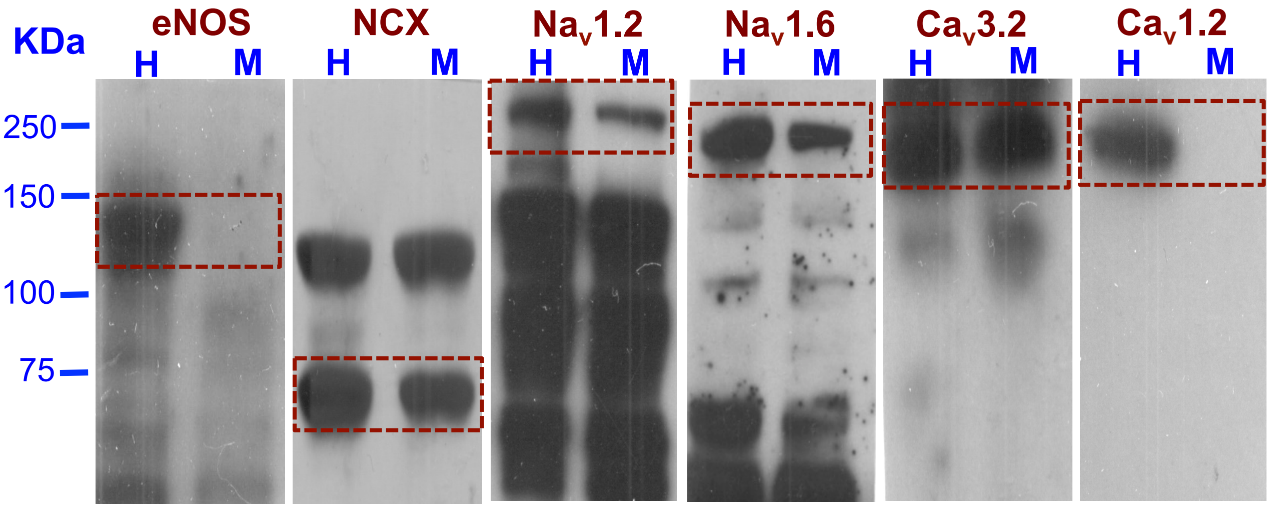

Supplementary Figure S4. Plasma membrane expression of Na$_v$1.2, Na$_v$1.6 and Ca$_v$3.2 channels in mesenteric resistance arteries. The presence of these channels in the plasma membrane was analyzed by biotinylation of cell surface proteins. Mesenteric arteries were perfused with 1 mM sulfosuccinimidyl-2-[biotinamido]ethyl-1,3-dithiopropionate for 40 min at 0.5 mL/min to label membrane proteins of the luminal surface of the vessels. Then, tissues were homogenized and proteins were precipitated with ice-cold acetone. The pellet was suspended in Tris-buffer, the biotinylated proteins were captured with streptavidin–agarose beads and separated by SDS-PAGE to be detected with specific antibodies. A representative Western blot of three independent experiments is shown. The presence of eNOS, Na^+-Ca^{2+} exchanger and the channels Na$_v$1.2, Na$_v$1.6, Ca$_v$3.2 and Ca$_v$1.2 was evaluated in the total tissue homogenized (**H**) and in the fraction of biotinylated-plasma membrane proteins (**M**). In addition to Na$_v$1.2, Na$_v$1.6 and Ca$_v$3.2 channels, eNOS and Ca$_v$1.2 were also analyzed as negative control to confirm that the biotin treatment did not label intracellular proteins of endothelial cells or plasma membrane proteins of smooth muscle cells. In addition, Na^+-Ca^{2+} exchanger (NCX) was used as a positive control for the detection of plasma membrane proteins of endothelial cells.

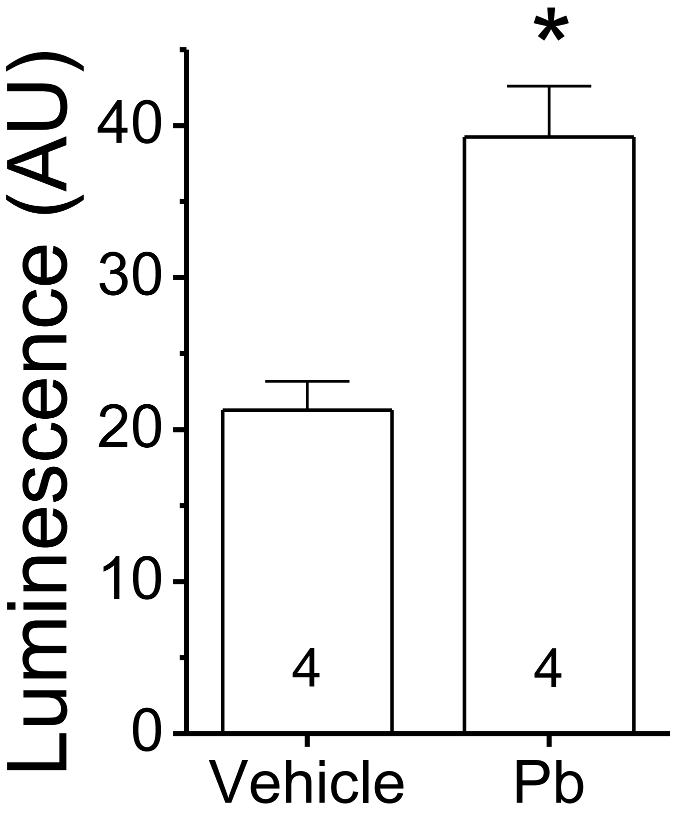

Supplementary Figure S5. Superoxide anion ($O_2^{\bullet-}$) production activated by probenecid (Pb). $O_2^{\bullet-}$ levels were measured in isolated mesenteric arterial beds of rats using the analysis by emitted light (ABEL®) assay, which is based on the intense luminescence emitted upon reaction of $O_2^{\bullet-}$ radicals with the prosthetic group of Pholasin, the photoprotein responsible for luminescence in the bivalve *Pholas dactylus*. After treating mesenteric vessels with the pannexin-1 channel blocker, probenecid, or its vehicle, 20 µL of perfusate samples were mixed with 100 µL of adjuvant-K™ solution and, following 1 min of equilibration, the reaction was initiated by the injection of 250 µL of Pholasin solution (10µg/mL). The resulting luminescence was immediately measured on a Turner TD20e luminometer (Promega). Adjuvant-K™ and pholasin solutions were prepared using the reconstitution buffer (Hank's balanced salt solution with 20 mM HEPES, pH 7.4) as indicated by the manufacturer (Knight Scientific Ltd.). Changes in $O_2^{\bullet-}$ formation are expressed in arbitrary units (AU). Numbers inside the bars indicate the n value. All measurements were made in duplicate. Values are means ± SEM. *, $P<0.05$ vs Vehicle by unpaired Student's t test.

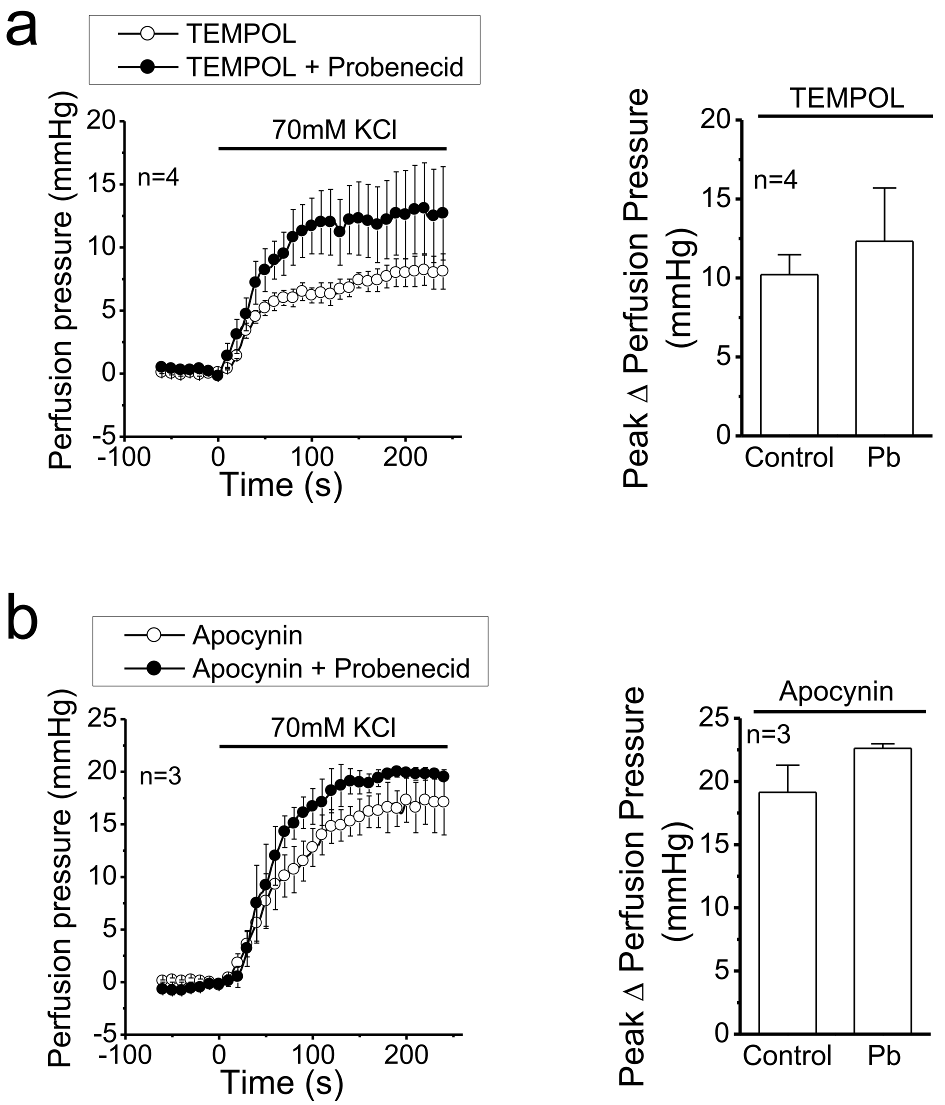

Supplementary Figure S6. The inhibition of the KCl-elicited vasoconstriction evoked by pannexin-1 channel blockade with probenecid (Pb) depends on a NADPH oxidase-initiated superoxide anion signaling. **a** and **b**, Effect of probenecid on the time course (Left) and maximum increase in perfusion pressure (Right) evoked by KCl (70 mM) in mesenteric arteries treated with TEMPOL (**a**), a superoxide scavenger, or apocynin (**b**), a NADPH oxidase inhibitor. Note that the reduction in the KCl-induced vasoconstriction observed after pannexin-1 channel blockade with probenecid (Supplementary Fig. S2) is fully prevented by TEMPOL (**a**) and apocynin (**b**). Horizontal bars indicate the period of KCl stimulation (Left) or the treatment applied (Right). Values are means ± SEM.
